# Congenital duplicated ureter-vagina anomalous anastomosis causing female urinary incontinence: a case report and literature review

**DOI:** 10.3389/fped.2025.1629410

**Published:** 2025-10-03

**Authors:** Shuxin Li, Hongliang Cao, Yueqiu Zhang, Jinghan Su, Tong Yang, Wei Wei, Xin Lian

**Affiliations:** ^1^ Department of Surgical Urology, The First Hospital of Jilin University, Changchun, China; ^2^ Department of Hepatobiliary and Pancreatic Surgery II, General Surgery Center, The First Hospital of Jilin University, Changchun, China

**Keywords:** heterotopic ureter, urinary incontinence, congenital ureterovaginal fistula, cystoscopy, ureteral bladder reimplantation

## Abstract

Ectopic ureter is a rare congenital anomaly, often challenging to diagnose due to atypical anatomical positioning and variable clinical presentations. Consideration should be given to the possibility of an ectopic ureteral orifice in young women presenting with persistent, unexplained urinary incontinence. This report details a 22-year-old nulliparous female presenting with persistent urinary incontinence. Imaging revealed left ureteral dilatation, with CT suggesting a possible connection between the distal ureter and vagina. Magnetic resonance imaging of the urinary system confirmed bilateral renal abnormalities with double ureteral malformations, hydronephrosis on the left side, and ectopic opening of the left ureter into the anterior vaginal wall, accompanied by a bicornuate uterus. Cystoscopy confirmed a double ureteric orifice on the right side and an ectopic left ureteral opening into the vagina. The patient underwent intraoperative cystoscopy, ureter cystostomy, and double-J stent placement. At the two-month post-operative follow-up, the patient's urinary incontinence symptoms and left-sided hydronephrosis with ureteral dilatation had completely resolved. Normal urinary function was restored, and the double-J stents were successfully removed via cystoscopy. This case underscores the need for heightened vigilance regarding congenital ectopic ureteral orifices in young women with no history of childbirth or surgery presenting with urinary incontinence. The combination of cystoscopy and imaging aids in definitive diagnosis, with surgical reimplantation yielding favorable outcomes. Early identification and intervention are crucial for improving prognosis.

## Introduction

Ectopic ureter is a rare congenital urological anomaly characterized by the abnormal positioning of the ureteral drainage opening, which is located outside the usual anatomical location. The prevalence of this condition is extremely low, typically ranging from 0.025% to 0.05% (1). Most ectopic ureters are associated with a dual collecting system in the urinary tract, featuring an abnormal number of ureters. These abnormal ureters may drain urine into the bladder or connect abnormally with other peripheral organs. Ectopic ureters are often detected in childhood, as they may be present from birth. Ectopic ureters may also be diagnosed at a later stage due to urinary tract infections, urinary incontinence, or other related symptoms (2, 3). The prevalence of ectopic ureters is significantly higher in women than in men (4). The cause of an ectopic ureter may involve abnormal embryonic development or a medical injury, both of which can result in an abnormal ureter position (5, 6). Additionally, ectopic ureters are frequently accompanied by other urinary tract malformations, such as duplicate kidneys or ureters. Clinical manifestations include urinary incontinence, urinary tract infections, and hydronephrosis (7, 8). Chronic hydronephrosis may impair renal function and, in severe cases, lead to renal insufficiency (9). Ectopic ureteral anomalies usually require surgical intervention. Surgical approaches to treating ectopic ureters include ectopic ureterostomy and possibly combined nephrectomy if the ipsilateral kidney is nonfunctioning. Suppose the patient's ureter and kidneys are functioning well. In that case, the less invasive option of ureteral bladder reimplantation, which restores the normal urinary flow pathway by dissecting and anastomosing the ureter to the bladder, can provide a better prognosis for the patient.

## Case presentation

**Figure 1:**
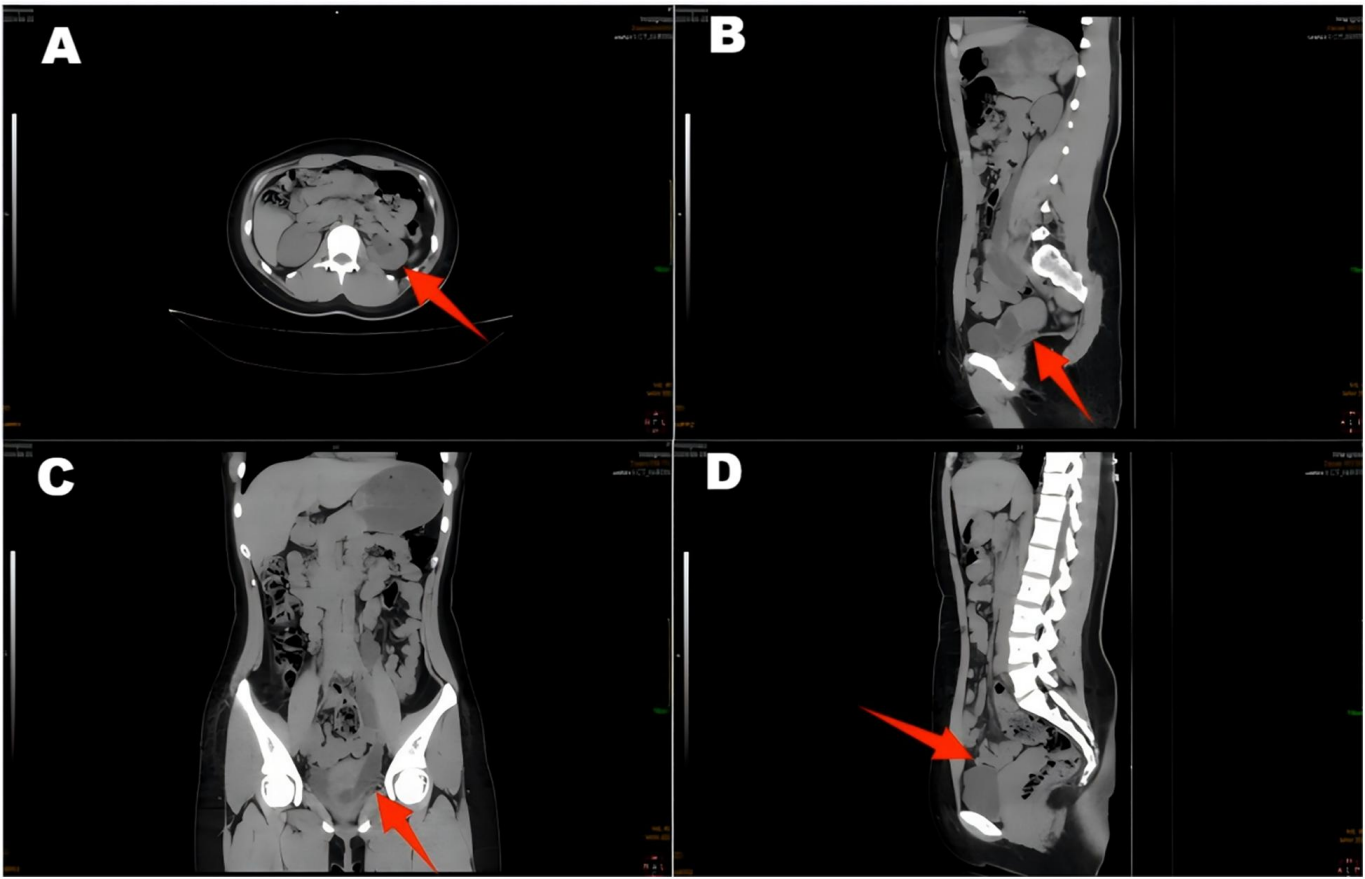
CT scan of the ureter shows hydronephrosis of the left renal pelvic ureter **(A,C,D)**, with a suspected terminal ureter connected to a cystic hypodense shadow of the vagina **(B)**.

**Figure 2:**
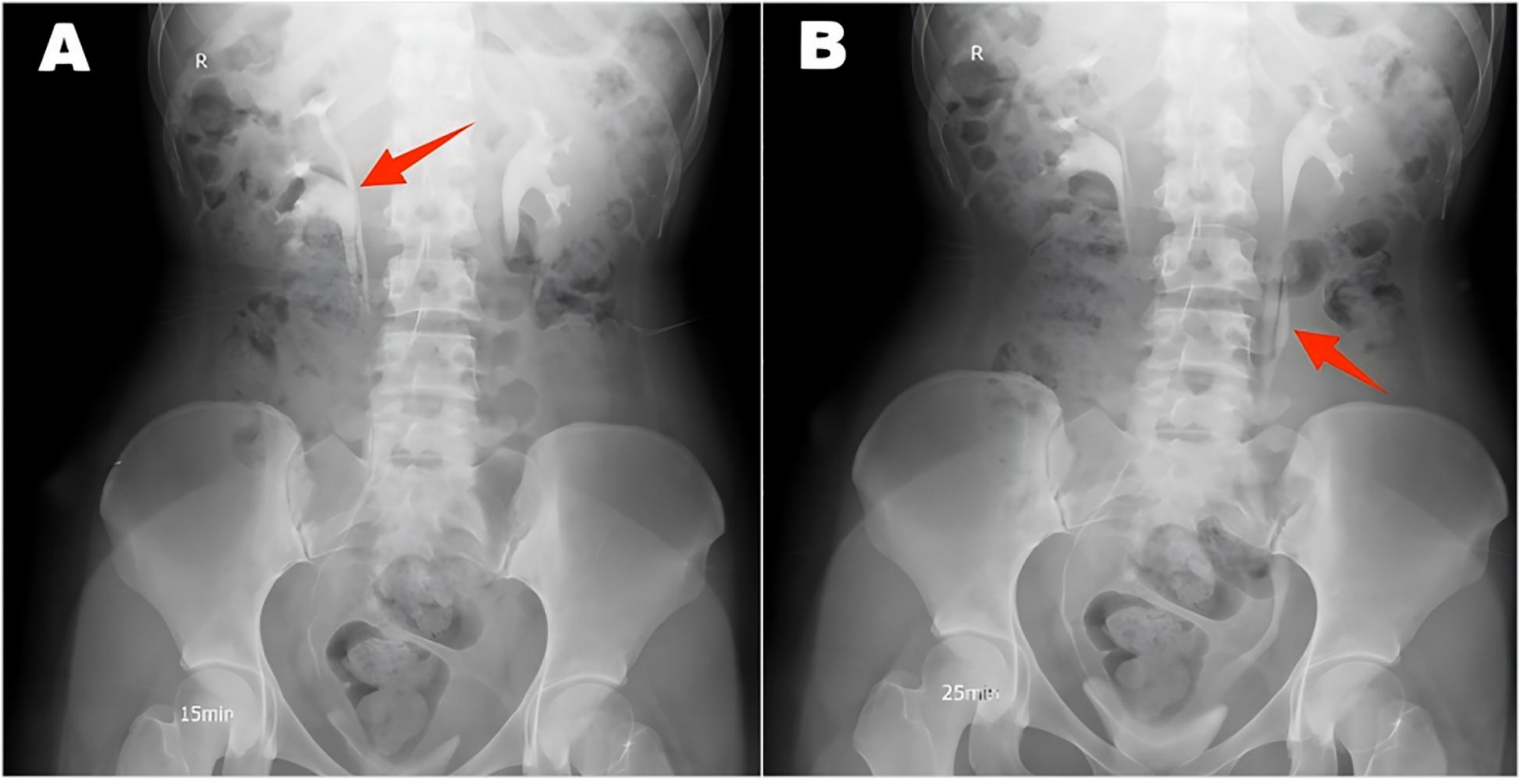
Intravenous urography revealed a double ureter on the right side **(A)** and a double ureter on the left side, which converged into a single ureter **(B)**.

**Figure 3:**
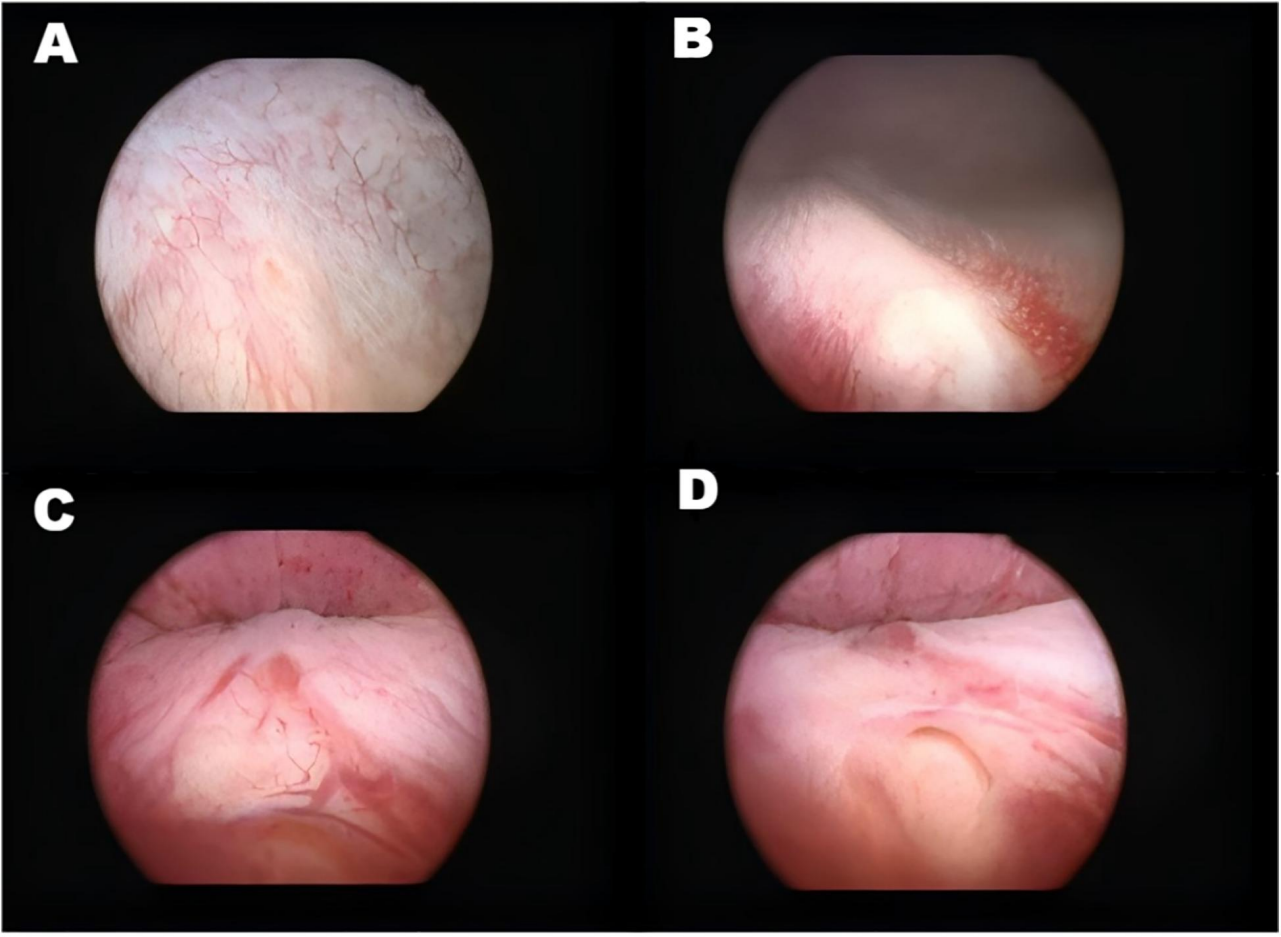
Illustrates various ureteral openings and related abnormalities. **(A)** Right sided orthotopic ureteral opening. **(B)** Right superior renal ureteral opening. **(C)** Abnormal mucosa in the urethra. **(D)** Left ectopic ureteral opening.

**Figure 4:**
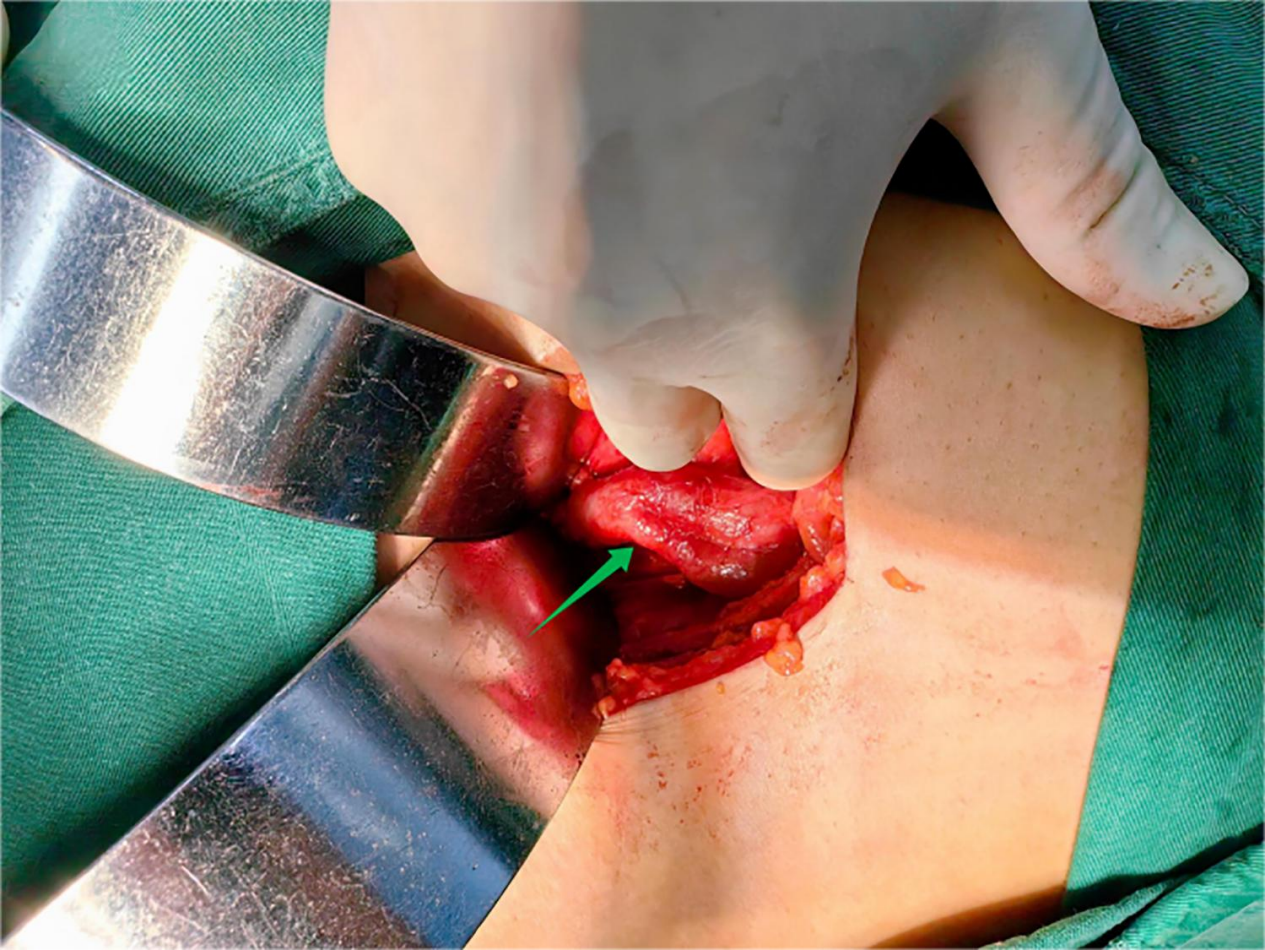
Two ureters converge to form a single ureter at the upper-middle end of the left side, and the lower end of the ureteral confluence joins the vagina.

A 22-year-old female patient presented with persistent urinary incontinence since infancy, spanning over two decades. The patient's perineal area remains persistently moist, requiring 3–4 diaper changes daily. The patient had no significant past medical history and no familial history of similar conditions. No history of surgery or childbirth at the time of consultation. The patient grew up in an orphanage after being abandoned as an infant. Due to this socially vulnerable background and lack of longstanding familial support, her condition was neither brought to medical attention nor formally evaluated in childhood or adolescence. Clinical examination revealed thickening of the external urethral orifice, with a suspected vaginal opening measuring approximately 0.7 centimeters in diameter. To determine the cause of the patient's long-standing urinary incontinence, we conducted a series of systematic imaging and endoscopic investigations. Initial multi-slice CT imaging revealed compensatory enlargement of the left kidney with hydronephrosis affecting the entire renal pelvis and ureter. A cystic, low-density lesion connecting the distal ureter to the anterior vaginal wall was identified, suggesting a possible ectopic ureteral opening into the vagina (Figure 1). Magnetic resonance imaging of the urinary system confirmed bilateral renal abnormalities with double ureteral malformations, hydronephrosis on the left side, and ectopic opening of the left ureter into the anterior vaginal wall, accompanied by a bicornuate uterus. Intravenous urography (IVU) further confirmed bilateral ureteral anomalies on the right side, whilst the dilated left ureter exhibited a bifurcated pattern in its middle and upper segments, ultimately converging near the bladder, thus ruling out complete duplication (Figure 2). Cystoscopy revealed two openings in the right ureter consistent with a duplicated ureteral anomaly. An ectopic opening of the left ureter was identified near the external urethral orifice on the anterior vaginal wall. In contrast, the ureteral opening on the ipsilateral side of the bladder trigone was absent (Figure 3). It should be noted that a limitation in this case was the absence of renal scintigraphy to assess left renal segmental function. This examination is crucial for identifying such congenital anomalies, serving as a key basis for guiding clinical decision-making and selecting the surgical approach. The patient underwent transurethral cystoscopy and left ureter cystostomy under general anesthesia. Intraoperatively, the left ureteral orifice was found to be ectopically located on the anterior vaginal wall near the external urethral orifice. Additionally, approximately 2 cm above the external iliac artery, the ureter exhibited thickened adhesions spanning about 8 cm in length (Figure 4). The definitively identified ectopic opening location precisely matched the preoperative cystoscopy localization, confirming the diagnosis of congenital ureterovaginal fistula. Ureter cystostomy was performed by creating a subcutaneous tunnel, excising the redundant ureter segment, and anastomosing it to the bladder mucosa, with a double-J stent placed. Postoperative urinary incontinence symptoms were completely resolved, and the patient was discharged successfully on the fourth postoperative day. During the two-month follow-up period, the patient reported having completely discontinued the use of urinary pads. Ultrasound follow-up revealed complete resolution of left hydronephrosis with no significant ureteral dilatation observed. After two months postoperatively, the double-J stent was removed via cystoscopy, and the patient's urinary function returned to normal.

## Discussion

An ectopic ureter is an anatomical abnormality of the urinary system characterized primarily by the end of the ureter not opening into the triangular region of the bladder, but draining into the non-triangular region of the bladder or an adjacent peripheral organ, such as the vagina, urethral vestibule, or rectum (4, 10). The vagina and urethra are common sites for ectopic ureteral openings (11). The prevalence of ectopic ureters in the overall population is approximately 0.025%–0.05% (2). Regarding gender distribution, the prevalence is significantly higher in females than in males, with a ratio of roughly 2–6 to 1 (2). The leading causes of the disease include congenital developmental anomalies and medically induced injuries, the latter of which are relatively rare, with most cases caused by congenital anomalies (4, 5). Individuals with a family history of ectopic ureters, exposure to abnormal environmental factors during pregnancy, and deletions and mutations in genes that inhibit the formation of anomalous ureters, such as the fetal BMP-4 gene, are risk factors that may increase the risk of developing ectopic ureters (12–14). The underlying cause of ectopic ureters is the failure of the ureteric bud to migrate normally to the bladder triangle during the embryonic period, resulting in its termination in an alternative location (4, 15). This anomaly usually occurs in the 4th–7th week of gestation and is associated with misalignment of the ureteric bud as it separates from Wolfe's canal (4, 15).

The most common clinical symptoms of ectopic ureter in women are persistent or intermittent urine leakage and recurrent urinary tract infections (16, 17). This is because the ectopic ureter bypasses the external urethral sphincter, interfering with its ability to control urine flow effectively and leading to abnormal urine distribution and an increased risk of urinary tract infections (18, 19). The clinical symptoms of male ectopic ureter are more insidious, mainly because male ectopic ureters are less likely to bypass the external urethral sphincter. Therefore, male urinary incontinence is less common (15, 20, 21). However, male patients may present with recurrent urinary tract infections or epididymitis, and the opening of the male ectopic ureter is most often located near the urethra or seminal vesicles in the prostate (15, 20). In women, the ectopic ureter may open in the vagina, which may be manifested as increased vaginal discharge or misdiagnosed as a gynecological disease (1, 22). If the ectopic ureter is combined with ureteral obstruction or hydronephrosis, low back pain may occur (22). Patients with ectopic ureters often have a combination of renal malformations, with 80% of female patients having ectopic ureters with double collecting systems, as well as other types of malformations, including dysplastic kidneys, ectopic kidneys, or atrophic kidneys (10, 16, 23). Infantile patients tend to present with symptoms of urinary incontinence or urinary tract infection or are diagnosed early due to prenatal ultrasound findings of hydronephrosis (24, 25). Some patients may exhibit no typical clinical symptoms and may be detected only incidentally in imaging studies (15, 23).

Although our case presented with typical clinical manifestations, the symptoms and diagnostic approaches for ectopic ureters vary significantly among individuals. To contextualize this case within a broader clinical framework, we reviewed recently published representative case reports (Table 1). Regarding diagnosis, imaging studies are crucial for confirmation, with ultrasound serving as an initial screening tool. However, literature consistently emphasizes the central role of magnetic resonance urography (MRU). For young patients with suspected complex congenital urological anomalies, MRU should be considered the initial imaging modality of choice due to its superior soft-tissue characterization, lack of ionizing radiation, and ability to provide detailed anatomical and functional information in a single examination (11, 28, 31–33). CT provides detailed three-dimensional imaging, and endoscopy allows for direct visualization of the opening location or surrounding urine accumulation (1, 11, 28, 34). Moreover, for patients with ectopic ureters, preoperative voiding cystourethrography is crucial for locating the ectopic ureteral orifice or assessing the presence of vesicoureteral reflux and bladder outlet obstruction (10, 35). Additionally, renal scintigraphy is indispensable for functional assessment, as it is crucial in determining whether to perform nephron-sparing or radical surgery (4, 36).

**Table 1:** Comparison of clinical manifestations, diagnosis, and treatment methods in published case reports of ectopic ureter.

Literature sources	Research size	Principal clinical manifestations	Key diagnostic methods	Surgical treatment methods
Qijing Wang et al. (1)	5 female cases	Gynecological disorders, urinary incontinence, pelvic pain	Pelvic ultrasound, urinary ultrasound, pelvic MRI, CT urography, urinary tract imaging	Urethrectomy/ureteral reimplantation/conservative observation
Abhijit Dhale et al. (4)	A 9-year-old female	Urinary incontinence	CT urography, DTPA scan	Right nephrectomy with urethrectomy
Nuru Bedru Hussen et al. (10)	A 24-year-old female	Urinary incontinence	Urinary system ultrasound, CT, pelvic MRI, and intravenous pyelogram	Nephroureterectomy
Ali Al-Smair et al. (15)	A 4-year-old female	Urinary tract infection and urinary incontinence	Ultrasound, intravenous pyelography, voiding cystourethrography	Urethrectomy
Lan Bu et al. (16)	13 cases (11 female, 2 male)	Persistent or intermittent urinary leakage (9 cases)/No leakage of urine (4 cases)	Unclear	Twelve surgical procedures performed/including one bilateral ureteral stent placement
Demisew Amenu et al. (23)	A 22-year-old woman	Urinary incontinence	Right renal ultrasound and contrast-enhanced CT	Uretero-bladder reimplantation
Barbara A Reinig et al. (26)	A 4-year-old female	Urinary tract infection	Static renal scintigraphy, renal dynamic scintigraphy, voiding cystourethrography, and urinary tract ultrasound	Ureteral reimplantation
Rizqia Abyaksa et al. (27)	A 19-year-old female	Urinary incontinence	Renal-ureteral-bladder ultrasound examination	Laparoscopic right ureteral hemicolectomy
Bogdan Toia et al. (28)	10 cases (9 female, 1 male)	Female urinary incontinence (9 cases), male lower urinary tract symptoms (1 case)	MRI	Partial nephroureterectomy/ectopic urethrectomy with bladder neck reconstruction/heminephrectomy and vesicostomy
Pritesh Jain et al. (29)	9 cases (6 female, 3 male)	Urinary incontinence, anorectal malformations, uterine abnormalities, bilateral absence of seminal vesicles with infertility, polycystic kidney hypoplasia, intrauterine ureteral drainage, renal failure, absence of the bladder trigone, and hypospadias.	Unclear	Ureteral reimplantation/ureteroureterostomy/upper pole nephrectomy/kidney transplantation
Eric S Chang et al. (30)	A 56-year-old female	Urinary incontinence and recurrent urinary tract infections	CT urography, cystoscopy, and right retrograde pyelography	Ectopic urethrectomy

Treatment options for ectopic ureters require comprehensive consideration of multiple factors, primarily including the degree of renal impairment, anatomical abnormalities, and the severity of clinical symptoms. For asymptomatic patients, no specific intervention is typically required, and regular follow-up observation is sufficient. In this case, despite the presence of a redundant right ureter with an opening not located in the bladder trigone, surgical resection is unnecessary since it has not caused any clinical symptoms. Standard surgical approaches for ectopic ureters include ectopic ureterostomy, ureter cystoplasty, ectopic urethrectomy, and ipsilateral nephrectomy (15, 30, 37, 38). A preoperative comprehensive evaluation is essential to determine the precise location of the ectopic ureter, renal morphology, and functional status, thereby enabling the development of an individualized surgical plan (16). In the comprehensive assessment of ectopic ureters, the integration of functional renal imaging techniques, such as dimercaptosuccinic acid (DMSA) or mercaptoacetyltriglycine (MAG-3) scintigraphy, is paramount (36, 39). Such imaging studies quantitatively assess the functional contribution of compromised nephrons and aid in distinguishing salvageable parenchymal tissue from segments with complete loss of function (36, 39). A significant limitation of our preoperative assessment was the absence of functional renal imaging studies. Such imaging techniques are crucial for quantifying functional disparities among affected renal segments, particularly in complex renal anomalies with underlying developmental abnormalities. Although CT supported the decision for ureteral reimplantation in this case and intravenous pyelography demonstrated preserved contrast excretion and intraoperative renal morphology, the lack of objective quantitative data remains a weakness in the evaluation. When imaging demonstrates minimal function in the relevant renal segment, the surgical strategy should shift toward pyeloureterectomy rather than reconstruction to eliminate nonfunctional tissue and sources of incontinence. For functional nephrons, ureter cystoplasty is the preferred approach, aiming to restore normal anatomy and maximize renal function preservation (37, 40). For cases of duplicated collecting systems with severe functional impairment in the upper renal segment, upper nephrectomy combined with urethrectomy is a common and effective treatment approach (15, 38, 41). Compared to urethrectomy or partial nephrectomy, ureter cystostomy offers advantages such as reduced trauma, greater preservation of nephrons, and lower risk of postoperative renal insufficiency. Additionally, uretero-ureteral anastomosis serves as a viable alternative, particularly for duplicated collecting systems, where the ectopic ureter can be reimplanted onto a normally positioned ureter (38, 42).

The surgical management of ectopic ureters has evolved with the adoption of minimally invasive techniques. Laparoscopic and robot-assisted surgery offer patients additional benefits, including reduced postoperative pain, shorter hospital stays, and improved cosmetic outcomes (43, 44). Furthermore, minimally invasive procedures are increasingly recognized as the standard treatment for many cases of ectopic ureter, particularly in pediatric patients (45, 46). These approaches are highly effective for nephrectomy, heminephrectomy, and even complex ureteral reimplantation in selected patients (43, 47, 48). In the present case, however, an open surgical approach was elected due to the intraoperative finding of significant peritureteral fibrosis and adhesions, which increased the complexity of dissection and the risk of inadvertent injury. The primary objective remained the successful resolution of incontinence and the preservation of renal function, which was achieved through ureteroneocystostomy.

## Conclusion

This case highlights the need for heightened vigilance regarding congenital ectopic ureteral orifices in young female patients presenting with persistent urinary incontinence, particularly in those without a history of childbirth or surgery. The condition can be definitively diagnosed through a combination of imaging studies and endoscopic examination. Surgical reconstruction to restore normal anatomical pathways significantly improves patients' quality of life and enhances the prognosis of renal function.

## Data Availability

The original contributions presented in the study are included in the article/Supplementary Material, further inquiries can be directed to the corresponding authors.
